# Cardiopulmonary Recovery After Maximal Exercise in Individuals with Neuromuscular Disease and Limited Mobility

**DOI:** 10.3390/jcm14124190

**Published:** 2025-06-12

**Authors:** Yair Blumberg, Constance de Monts, Samuel Montalvo, Whitney J. Tang, Sally Dunaway Young, Nathan Hageman, Fabian Sanchis-Gomar, Euan A. Ashley, David Amar, Jonathan Myers, Matthew T. Wheeler, John W. Day, Tina Duong, Jeffrey W. Christle

**Affiliations:** 1 Division of Cardiovascular Medicine, Department of Medicine, Stanford University, Stanford, CA 94305, USA; yair.blumberg@gmail.com (Y.B.); sangofa@stanford.edu (F.S.-G.);; 2 Division of Neurology and Neurological Sciences, Department of Medicine, Stanford University, Stanford, CA 94305, USAtrduong@stanford.edu (T.D.); 3 Wu Tsai Human Performance Alliance, Stanford University, Stanford, CA 94305, USA; 4 Department of Health Sciences, University of Burgos, 9059 Burgos, Spain; 5 Division of Cardiology, Veterans Affairs (VA), Palo Alto Health Care System, Palo Alto, CA 94304, USA

**Keywords:** neuromuscular disease, cardiopulmonary exercise testing, exercise recovery, 50% recovery, overshoot

## Abstract

**Background:** Individuals with neuromuscular diseases (NMDs) have low physical activity levels and an increased risk of cardiovascular and pulmonary diseases. Respiratory gas kinetics obtained during cardiopulmonary exercise testing (CPET) may provide valuable insights into disease mechanisms and cardiorespiratory fitness in individuals with NMD. Recovery from exercise is an important marker of exercise performance and overall physical health, and impaired recovery is strongly associated with poor health outcomes. This study evaluates recovery metrics in individuals with NMD after performing maximal exertion during CPET. **Methods:** A total of 34 individuals with NMD and 15 healthy volunteers were recruited for the study. CPET was performed using a wearable metabolic system and a wheelchair-accessible total body trainer to peak exertion. Recovery metrics assessed were (i) the time to reach 50% O_2_ recovery compared with peak exercise and (ii) the ratios of ventilation and respiratory gases between peak exercise and the highest values observed during recovery (overshoot). **Results:** The NMD group had a significantly longer time to reach 50% O_2_ recovery (T1/2 VO_2_: 105 ± 43.4 vs. 76 ± 36.4 s, *p* = 0.02), lower respiratory overshoot (17.1 ± 13.0% vs. 28.8 ± 9.03%), and lower ventilation/VO_2_ (31.9 ± 28.3 vs. 52.2 ± 23.5) compared to the control group. **Conclusions:** This study observes significantly impaired recovery metrics following peak exercise in individuals with NMD compared to controls. These insights may improve the understanding of exercise recovery and mechanics, thus improving prognostication and optimizing exercise prescriptions for individuals with NMD.

## 1. Introduction

Neuromuscular diseases (NMDs) encompass a spectrum of conditions with varying degrees of muscle weakness, fatigue, and impairment in movement. NMD has a significant impact on an individual’s ability to perform physical activities due to progressive muscle weakness, contributing to a sedentary lifestyle that further exacerbates their symptoms [1]. In addition, the reduced physical activity level increases the risk of cardiovascular and pulmonary diseases, as well as muscle atrophy [1,2]. Exercise recommendations in NMD have been varied, reflecting the diverse evidence base and incomplete understanding of the mechanisms underlying exercise recovery and response in this population [3,4].

Recent evidence suggests that aerobic exercise is a safe and effective treatment option for most individuals with NMD. Still, there remains a lack of clarity regarding exercise prescription and recovery time, and it remains unclear what metrics are most important in determining positive responses to exercise interventions [5,6]. In a recent survey of neuromuscular clinicians, 81% of respondents believed that exercise should be integrated into care protocols. Nevertheless, a primary barrier identified was insufficient knowledge regarding accurately prescribing and monitoring exercise responses in this population [7].

Several physiological processes are involved in exercise recovery, including removing metabolic waste products, repairing tissues, and replenishing energy [8]. The effectiveness and duration of recovery depend on factors such as exercise intensity, individual fitness level, and underlying health conditions [9]. Addressing factors that may impair recovery, such as chronic inflammation or metabolic disorders, may improve exercise performance and overall physical health [8].

Cardiorespiratory fitness (CRF) is a critical metric determining an individual’s ability to use oxygen efficiently during physical exertion. Cardiopulmonary exercise testing (CPET) is the gold-standard method for evaluating CRF, which measures variables such as peak oxygen consumption (VO_2_), ventilation (VE), and carbon dioxide output (VCO_2_) during incremental exercise [10,11]. Recent work has focused on analyzing the recovery patterns following CPET, as this may provide valuable insights into the underlying mechanisms of various diseases; recovery patterns can provide important insights into understanding cardiopulmonary training effects in different chronic conditions [12,13,14,15,16,17].

While heart rate (HR) recovery is an established diagnostic and prognostic indicator for cardiovascular disease, understanding of gas exchange recovery kinetics remains limited. Yet gas exchange recovery kinetics is hypothesized to provide complementary information and may improve prognosis and diagnosis related to cardiorespiratory disease [14,18,19]. Recent studies have demonstrated that individuals with diabetes and chronic heart failure (CHF) exhibit slower VO_2_ recovery after exercise. Specifically, CHF individuals require more time to return to baseline VO_2_ values, and the severity of the disease negatively impacts their VO_2_ recovery [15,20,21,22,23].

For individuals with NMD, exercise recovery can be compromised by both cardiorespiratory difficulties and muscle impairment, resulting in mitochondrial abnormalities, metabolic dysfunction, and inflammatory responses to exercise [24]. These factors can lead to delayed-onset muscle soreness, prolonged fatigue, and impaired muscle regeneration, thus highlighting the need to consider exercise-specific muscle responses in treatment strategies.

In this study, we aimed to evaluate the differences in physiological recovery variables between individuals with NMD and healthy controls.

## 2. Materials and Methods

Patients with NMD were prospectively recruited as a sample of convenience from patients being treated at the Neuroscience Health Center at Stanford University from 2023 to 2024. All had limited mobility, which was defined as the inability to perform cycle ergometry or treadmill ambulation safely. Individuals were excluded if they could not provide informed consent/assent, had engaged in regular treadmill or upright cycle ergometry exercise within the last six months, or were deemed unable to exercise vigorously by the study physicians. Participants in the control group were recruited from the local community and did not have medical diagnoses that would prohibit maximal exercise testing performance, and efforts were made to match for age and sex. This study was approved by the Institutional Review Board at Stanford University (code: #23888, date: 11 December 2024). All research staff were trained in good clinical practice, and all participants provided written informed consent or assent before any study procedures.

Thirty-six participants with NMD were initially included in this study; four patients were excluded due to technical errors that resulted in incomplete and/or unreliable CPET data (two with myotonic dystrophy, one with Duchenne muscular dystrophy, and one with limb–girdle muscular dystrophy). Thirty-two patients with NMD and fifteen controls were included. The participants in the study did not have any documented comorbidities such as heart failure, obstructive sleep apnea, hypertension, or diabetes mellitus.

### 2.1. Cardiopulmonary Exercise Testing (CPET)

CPET was performed using a CosMed K5 wearable metabolic system (COSMED USA Inc, Concord, CA, USA) and a Keiser wheelchair-accessible total body trainer (Keiser Corporation, Fresno, CA, USA) that allowed individuals to use lower limb strength in an elliptical pattern and upper limb strength in a push–pull pattern. The CPET protocol used in the current study has been described previously [10].

CPET was performed by clinical exercise physiologists and physical therapists and monitored by a physician familiar with the individuals and the study protocols. Respiratory gas exchange data were collected on a breath-by-breath basis and analyzed after applying a 30 s rolling average filter (sampled every 10 s) and were calculated as the highest 30 s average during the last phase of the CPET at the point of peak ventilation. The percentage of age-predicted peak VO_2_ reached by participants was calculated using the Fitness Registry and Importance of Exercise National Database (FRIEND) Registry protocol [25]. Peak HR (bpm), rating of perceived exertion (RPE 6–20; Borg Perception AB, Akersberga, Sweden), and workload (W) were calculated as the maximal number recorded during exercise. The VE/VCO_2_ slope was calculated for the entire exercise period [25].

Recovery was defined as the period from the end of exercise until the cessation of the measurement (a five-minute period). Respiratory gases (VO_2_ and VCO_2_) were analyzed as a percentage change from the time of exercise cessation to the time to reach 50% (T1/2) of peak VO_2_ during recovery. The overshoot percent changes from peak values were calculated for the following metrics: respiratory exchange ratio (RER; calculated as the ratio of VCO_2_/VO_2_), ventilatory equivalents for oxygen (VE/VO_2_) and carbon dioxide (VE/VCO_2_), and partial pressures of oxygen (PETO_2_). These were calculated as the magnitude of the overshoot (i.e., the maximum percentage increase from peak) occurring during the five minutes of recovery. The 50% respiratory recovery metrics—volume of exhaled oxygen (VO_2_), volume of exhaled carbon dioxide (VCO_2_), and ventilation (VE)—were defined as the time required to reach 50% of the peak.

### 2.2. Statistical Analysis

CPET variables of interest were extracted from the metabolic cart and filtered as described above. Data were analyzed using the R statistical programming language in Rstudio IDE (R 4.3.2). Before conducting inferential statistics, data normality was assessed using the Shapiro–Wilk test, which indicated a non-normal distribution of all data. Consequently, we adopted a non-parametric test approach. We applied a Wilcoxon rank-sum test for differences between groups (NMD vs. control), setting the statistical significance at an alpha level of 0.05. The descriptive statistics, including mean and standard deviation and median and interquartile range (IQR), were calculated and are presented in Section 3 in the text and accompanying tables.

## 3. Results

Table 1 presents the demographic statistics of the 32 individuals by disease group. There were no significant differences in age, height, body weight, or BMI between the control and NMD groups.

Table 2 shows significant differences between the control and NMD groups during CPET. Peak workload was significantly higher in the control group compared to the NMD group (172 [140, 282] W vs. 54.5 [10.0, 168] W; *p* < 0.001). Absolute and relative peak VO_2_ were significantly higher in the control group (2.37 [1.39, 4.07] vs. 1.37 [0.532, 3.04] L/min, 33.0 [19.9, 54.3] kg/mL/min vs. 19.5 [10.8, 34.2] mL/kg/min; both *p* < 0.001). Similar results were observed regarding the percentage of age-predicted peak VO_2_ (104 [72.0, 182]% vs. 52.4 [26.3, 124]%; *p* < 0.001). Peak RER and peak HR were also significantly higher in the control group (1.13 [1.01, 1.38] vs. 1.06 [0.810, 1.39]; *p* < 0.02 and 173 [157, 193] bpm vs. 150 [95.0, 192] bpm; *p* < 0.001). The surrogate marker for stroke volume, O_2_ pulse, calculated as the ratio of VO_2_ to HR and expressed as the volume of oxygen consumed with each cardiac contraction, was significantly higher in the control group, with a median value of 14.9 [10.5, 23.5] mL/bpm, compared to the NMD group, which had a median value of 8.70 [2.80, 19.8] mL/bpm (*p* < 0.001).

**Figure 1:**
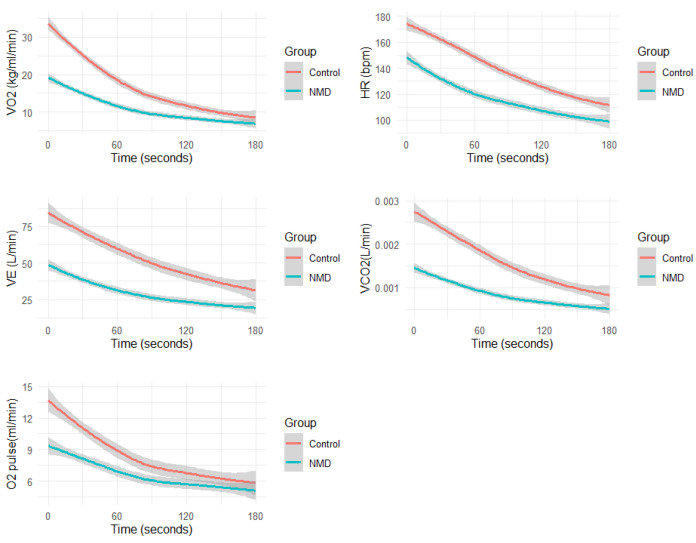
Average physiological responses during recovery: oxygen consumption (VO_2_), carbon dioxide production (VCO_2_), minute ventilation (VE), oxygen consumption per heart rate (VO_2_/HR), and heart rate (HR).

As shown in Table 3 and Figure 1, physiological recovery parameters after CPET differed significantly between the control group and the NMD group. For T1/2 VO_2_, the control group had a faster O_2_ recovery (60.0 [30.0, 180] s) versus the NMD group (90.0 [50.0, 210] s; *p* = 0.02). The recovery time for the O_2_ pulse (T1/2 O_2_ pulse) was also significantly longer in the NMD group compared to the control group (165 [60.0, 330] s vs. 90.0 [30.0, 260] s; *p* < 0.001).

**Figure 2:**
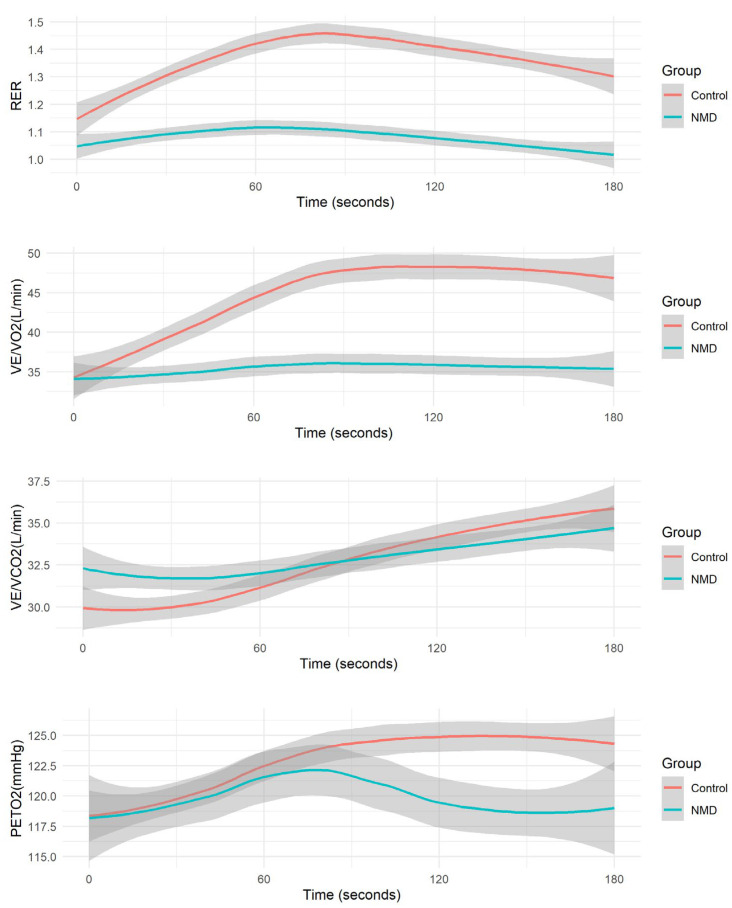
Average overshoot metrics during recovery: respiratory exchange ratio (RER), ventilation to oxygen consumption ratio (VE/VO_2_), ventilation to carbon dioxide production ratio (VE/VCO_2_), and partial pressure of oxygen (PETO_2_).

Significant differences were observed between the groups for the overshoot parameters (Figure 2). The control group exhibited a mean overshoot of RER of 30.0 [13.7, 43.4]%, significantly higher than the NMD group’s mean of 16.2 [0.730, 53.6]% (*p* < 0.001). The mean overshoot of VE/VO_2_ was also higher in the control group: 49.5 [22.5, 99.8]% vs. 30.5 [1.85, 124]%, respectively (*p* = 0.01). A difference was also noted in the overshoot of VE/VCO_2_, with the control group having a mean of 28.9 [3.03, 59.6]% vs. the NMD group’s mean of 15.5 [0.338, 109]% (*p* = 0.04).

## 4. Discussion

This exploratory study investigated recovery metrics following peak exercise in a relatively small sample of individuals with NMD. Established metrics for CPET testing, including peak VO_2_ and ventilatory efficiency (VE/VCO_2_ slope), have been used as indicators of CRF. However, physiological responses during recovery provide additional insights into fatigability, exercise intolerance, and muscle recovery mechanisms. Although HR has been a well-established method to assess recovery, understanding gas exchange kinetics may provide more insight into central versus peripheral muscular determinants for aerobic capacity [18]. A study by Cohen-Solal et al. found that delayed VO_2_ recovery may explain the delayed replenishment of muscle energy stores [26]. This deficiency in energy stores has been associated with poor aerobic efficiency.

Recent research underscores the significance of overshoot metrics during recovery as metabolic and cardiovascular function markers across various patient populations [7,14,15,16,21]. Vecchiato et al. demonstrated that elevated RER overshoot in young CHD patients reflected impaired oxygen delivery and exercise intolerance [15,16]. In contrast, low RER overshoot during recovery in patients with heart failure with reduced ejection fraction (HFrEF) is associated with poorer clinical outcomes [15,16]. Similarly, studies by Takayanagi and Patti et al. showed that reduced overshoot metrics, particularly RER, correlate with the severity of heart failure [14,21]. We observed that patients with NMD and low mobility showed distinct impairments in recovery, particularly in overshoot parameters, especially RER overshoot, compared with controls. Although there are some similarities between patients with NMD and CVD, which may suggest cardiovascular dysfunction and reduced cardiac output (especially lower peak HR and oxygen pulse), the larger differences in peak workload and oxygen consumption seen in NMD are likely at least partially due to a combination of skeletal muscle dysfunction from the underlying neuromuscular disease and deconditioning. Although the current study did not include any participants with CV comorbidities for comparison, these results do support that there is a distinct neuromuscular component which is related to decreased exercise capacity and abnormal CPET results, which is in agreement with the observations of previous studies [27]. These findings highlight the value of RER overshoot as a key metric for understanding recovery physiology and tailoring interventions for diverse clinical populations, including those with neuromuscular diseases. Future studies should explore its prognostic relevance and potential role in guiding individualized rehabilitation strategies for NMD patients.

Our study findings reveal substantial differences in recovery exercise variables between individuals with NMD and controls. The peak exercise variables highlight significant distinctions in exercise capacity between the two groups. Notably, the control group demonstrated higher peak workload, peak VO_2_, percentage of age-predicted peak VO_2_, peak HR, and O_2_ pulse versus the NMD group. These findings coincide with symptoms in individuals with NMD due to impaired muscle function and other factors associated with functional limitations [5,10,27,28].

Regarding recovery metrics, the NMD group exhibited longer VO_2_ recovery time (T1/2 VO2) compared to controls. They also showed lower overshoot values for RER and VE/VO2 ratio, likely driven by CO_2_ output and decreased plasma pH. These delayed recovery patterns are similar to those observed in patients with CVD [14,15,26], likely reflecting impaired muscle energy replenishment and cardiovascular limitations restricting oxygen delivery and utilization [26].

While delayed VE and VCO_2_ recovery reflect impaired cardiac function for individuals with CHF [21,26,29], the absence of differences in the T1/2 of VE and VCO_2_ between the control and NMD groups suggests that recovery limitations in NMD may stem from distinct peripheral mechanisms rather than cardiovascular limitations to oxygen delivery; the prolonged oxygen uptake kinetics likely stem primarily from skeletal muscle impairments that restrict cellular energy replenishment after peak exertion. However, this difference from cardiac patients may depend on the specific NMD subtype, as some neuromuscular disorders have associated cardiac involvement [24,30]. Thus, while some recovery metrics such as T1/2 VO_2_ mirror patterns seen in CHF, preserved VE and VCO_2_ recovery indicate the participation of different pathological processes centered in the muscle itself. However, cardiovascular comorbidities in certain NMD subpopulations could display hybrid cardiac–muscular recovery phenotypes [14,26].

Future studies should explore the reasons behind limitations in oxygen utilization and identify whether they stem from muscle-level factors or are associated with factors related to ventilatory insufficiency. Including CPET recovery parameters among individuals with NMD will enhance our ability to stratify NMD cohorts with known cardiovascular deficits compared to those without cardiorespiratory complications, providing valuable insights into potential impairments in muscle metabolomics or cardiorespiratory responses. Additionally, integrating dual-energy X-ray absorptiometry (DEXA) will enhance our understanding of the interplay between muscle and cardiopulmonary impairments, enabling a more individualized approach to exercise prescriptions. These insights into recovery have the potential to enhance the measurement of function and fatigue following physical exertion and inform recommendations for improving recovery responses in NMD. With additional CPET data in NMD, we may be better equipped to differentiate between individuals based on factors such as ventilatory insufficiency or muscle composition.

## 5. Conclusions

Many individuals with NMD experience exertional impairments characterized by fatigue, exercise intolerance, breathlessness, limited ability for sustained activity, and reduced engagement in an active lifestyle [5,6,31]. Analyzing cardiorespiratory recovery responses from maximal exercise offers insights into peripheral and central mechanisms that contribute to reduced oxygen utilization. A thorough grasp of recovery metrics enhances precision in tailoring home exercise programs and understanding rest, recovery, and fatigability. Improved comprehension of these metrics may offer a novel approach to assessing improvements in CRF, serving as a valuable endpoint for measuring exercise capacity and function for individuals with NMD.

## 6. Limitations

The current study, as in many studies in NMD, was limited by a relatively small and heterogeneous sample of convenience. Nonetheless, the sample represents a real-world example of individuals with NMD, reflecting those who would be seen in a typical NMD clinic. Second, it is likely that a five-minute duration for recovery from peak exercise may not be long enough to recover adequately for some individuals with NMD. In practice and for future studies, a longer (10–15 min) duration could be warranted and may provide more information about the physiology of recovery. Another limitation of our analysis was the inability to investigate recovery metrics over time. In addition, we lacked DEXA scans, which would have provided valuable insights into body composition, particularly muscle mass. Integrating DEXA scans with our CPET data could have elucidated how variations in muscle mass impact exercise performance, allowing for a more comprehensive understanding of exercise recovery among individuals with differing body compositions. Finally, we were unable to perform echocardiography, which would have perhaps provided valuable insight into the nature of exercise limitation. Therefore, an underlying cardiac involvement cannot be excluded.

## Figures and Tables

**Table 1:** Demographic characteristics of participants.

	Control (N = 15)	NMD (N = 32)	*p*
Age (yrs)			
Median [Min, Max]	33.3 [22.7, 70.7]	43.9 [11.8, 78.0]	0.46
Height			
Median [Min, Max]	173 [154, 193]	170 [137, 196]	0.05
Body Weight (kg)			
Median [Min, Max]	64.9 [54.0, 96.6]	67.1 [42.2, 147]	0.86
BMI (kg/m^2^)			
Median [Min, Max]	22.9 [18.5, 30.3]	23.2 [15.3, 42.6]	
Resting HR (bpm)			
Median [Min, Max]	69 [60, 81]	70 [58, 84]	0.57
Disease			
DM1	-	7 (21.9%)	
DM2	-	1 (3.1%)	
DMD	-	1 (3.1%)	
Dysferlinopathy	-	1 (3.1%)	
Dystrophinopathy	-	1 (3.1%)	
FSHD	-	10 (31.3%)	
LGMD	-	1 (3.1%)	
Pompe	-	3 (9.4%)	
SMA3	-	7 (21.9%)	

DM1, myotonic dystrophy type 1; DM2, myotonic dystrophy type 2; DMD, Duchenne muscular dystrophy; FSHD, facioscapulohumeral muscular dystrophy; LGMD, limb–girdle muscular dystrophy; SMA3, spinal muscular atrophy type 3.

**Table 2:** Peak exercise values during cardiopulmonary exercise testing (CPET) by group.

	Control (N = 15)	NMD (N = 32)	*p*
Peak Workload (Watt)			
Median [Min, Max]	172 [140, 282]	54.5 [10.0, 168]	<0.001
Peak VO_2_ (L/min)			
Median [Min, Max]	2.37 [1.39, 4.07]	1.37 [0.532, 3.04]	<0.001
Peak VO_2_ (mL/kg/min)			
Median [Min, Max]	33.0 [19.9, 54.3]	19.5 [10.8, 34.2]	<0.001
Predicted Peak VO_2_ (%)			
Median [Min, Max]	104 [72.0, 182]	52.4 [26.3, 124]	<0.001
VO2 at AT (mL/kg/min)			
Median [Min, Max]	17.0 [14.4, 25.1]	11.2 [8.24, 11.42]	<0.001
Peak RER (no unit)			
Median [Min, Max]	1.13 [1.01, 1.38]	1.06 [0.810, 1.39]	0.02
Peak Heart Rate (bpm)			
Median [Min, Max]	173 [157, 193]	150 [95.0, 192]	<0.001
Predicted Heart Rate (%)			
Median [Min, Max]	93.8 [88.1, 111]	86.3 [62.7, 108]	<0.001
O_2_ pulse (mL/bpm)			
Median [Min, Max]	14.9 [10.5, 23.5]	8.70 [2.80, 19.8]	<0.001
VE VCO_2_ Slope (no unit)			
Median [Min, Max]	32.2 [27.0, 43.3]	36.7 [27.4, 53.8]	0.09
VT1 VE VCO_2_ Slope (no unit)			
Median [Min, Max]	24.2 [16.4, 29.0]	27.6 [21.7, 43.5]	0.06
VT2 VE VCO_2_ Slope (no unit)			
Median [Min, Max]	26.9 [24.1, 30.4]	29.8 [25.6, 50.4]	0.01

RER, respiratory exchange ratio; VCO_2_, carbon dioxide production: tidal ventilation; VT: ventilatory threshold.

**Table 3:** Physiological recovery parameters during cardiopulmonary exercise testing (CPET); 1/2 refers to the time to reach 50% of the maximum.

	Control (N = 15)	NMD (N = 32)	*p*
T1/2 VO_2_ (second)			
Median [Min, Max]	60.0 [30.0, 180]	90.0 [50.0, 210]	0.02
T1/2 VE (second)			
Median [Min, Max]	120 [30.0, 230]	120 [40.0, 280]	0.94
T1/2 VCO_2_ (second)			
Median [Min, Max]	90.0 [50.0, 190]	100 [40.0, 180]	0.54
T1/2 O2 Pulse (second)			
Median [Min, Max]	90.0 [30.0, 260]	165 [60.0, 330]	<0.001
Overshoot of RER (%)			
Median [Min, Max]	30.0 [13.7, 43.4]	16.2 [0.730, 53.6]	<0.001
Overshoot of VE/VO_2_ (%)			
Median [Min, Max]	49.5 [22.5, 99.8]	30.5 [1.85, 124]	0.01
Overshoot of VE/VCO_2_ (%)			
Median [Min, Max]	28.9 [3.03, 59.6]	15.5 [0.338, 109]	0.04
Overshoot of PETO_2_ (%)			
Median [Min, Max]	8.55 [3.23, 14.0]	4.76 [0.813, 62.4]	0.08

1/2 refers to the time to reach 50% of the maximum.

## Data Availability

The original contributions presented in this study are included in the article. Further inquiries can be directed to the corresponding author.
